# The relationship between menopausal symptoms and burnout. A cross-sectional study among nurses

**DOI:** 10.1186/s12905-019-0847-6

**Published:** 2019-11-27

**Authors:** Daniela Converso, Sara Viotti, Ilaria Sottimano, Barbara Loera, Giorgia Molinengo, Gloria Guidetti

**Affiliations:** Department of Psychology, University of Turin, Via Giuseppe Verdi 10, 10124 Turin, Italy

**Keywords:** Menopause, Professional burnout, Psychosocial factors, Workplace, Nurses.

## Abstract

**Background:**

Despite the growing presence of menopausal women in workplaces, studies aimed at exploring the link between menopausal symptoms and job well-being are scarce. In the interest of addressing this gap, the present study aimed to explore whether menopausal symptoms might contribute to increased levels of burnout and whether this relationship can be moderated by social or personal resources.

**Method:**

The study design was cross-sectional and non-randomized. Ninety-four menopausal nurses completed a self-report questionnaire including scales aimed at measuring menopausal symptoms, burnout, social (i.e., support from superiors and colleagues) and personal (i.e., self-efficacy, resilience, and optimism) resources. Moderated regression analyses were performed to test study hypotheses.

**Results:**

Whereas menopausal symptoms were associated significantly with emotional exhaustion, no social or personal resources were found to moderate this relationship. Regarding depersonalization, our study indicated that it was affected by menopausal symptoms only among nurses who reported low social support (from superiors and colleagues), optimism, and resilience.

**Conclusion:**

The present study highlights the importance of organizations that employ a growing number of menopausal women to seek solutions at the individual and social levels that help these women deal with their menopausal transition while working.

## Background

Menopause is a complex physiological process that marks the end of the reproductive phase of a woman’s life [1], entailing a variety of symptoms often attributed to hormonal changes. However, other factors, such as health conditions or lifestyle choices, also can affect menopausal symptoms [2]. Typical examples of menopausal symptoms include hot flashes, sleep disturbances, decreased physical strength, mood changes, and bladder irritability. These symptoms might vary in terms of incidence and intensity across individuals and various phases of the menopausal process (i.e., pre-, peri-, or post-menopausal periods [2]). Symptoms appear, on average, between ages 48 and 55, with menopausal transition typically lasting four to 8 years [3].

The increasing presence of women in the workplace and the aging of the workforce have elicited growing interest in the link between menopause and work [4, 5]. In European countries, employment rates for older workers (ages 55–64) increased nearly 10% between 2000 and 2010 [6]. In 2000, the employment rate for women ages 55–64 was 27.4%, but by 2010, the rate increased to 38.8% [6]. Moreover, the number of those age 65 and over is expected to increase 20% by 2020 [7]. Thus, menopausal women will become even more common in the workplace.

In the nursing context, menopause at work is now a relevant issue that will become urgent. The Italian public-health sector particularly demonstrates this trend: Of the 273,267 nurses fully employed, 77.2% were women (women = 211,207, men = 62,207 [8]), and about 21% of these women were ages 45–49, 16.5% were 50–54, and 13.2% were 55 or older [8].

The literature has highlighted how menopausal symptoms negatively affect the quality of women’s personal lives, lessening levels of general subjective well-being [9, 10]. Studies in the occupational-medicine field provide evidence of how certain aspects of the physical work environment (e.g., temperature) exacerbate menopausal symptoms [11]. Other studies in the economic field have highlighted direct and indirect costs from a lack of organizational support for menopausal women in terms of increased absenteeism, presenteeism, and medical checkups [12].

On the other hand, previous literature that has examined menopause from an occupational health psychology (OHP) perspective is in its infancy [13], with a few pioneering studies having examined the relationship between some dimensions of work experience (e.g., work ability [14]) and menopausal symptoms. More recent studies have provided evidence for the association among certain psychosocial work-related factors (e.g., social support, job autonomy) and menopausal symptoms [15–17]. However, the mechanisms that link menopausal status and job well-being (if any) remain unclear, and no study is available regarding factors that might promote the sustainability of work among women during their menopausal transition.

Considering the large presence of women within the menopausal age range working in nursing settings [8], the present study chose to focus on nurses. It has been well-established in extant literature that nurses are at higher risk of developing burnout [18, 19], so the present study aimed to explore whether menopausal symptoms might contribute to increasing levels of burnout and whether social or personal resources can moderate this relationship.

### Relationship between menopause and burnout

Professional burnout is a psychological response to chronic work-related stress of an interpersonal and emotional nature that appears in professionals working directly with clients, patients, or others [20]. Many symptoms can be observed during the development of burnout, such as reduced personal accomplishment, guilt, or work-related anxiety [21, 22]. However, many scholars [23, 24] have noted two as being “core dimensions” that characterize this syndrome: emotional exhaustion and depersonalization. The exhaustion component represents the basic individual stress dimension of burnout [25]. It refers to feelings of being overextended and depleted of one’s emotional and physical resources [20]. Depersonalization refers to interpersonal relationships, denoting negative, callous, or excessively detached attitudes toward care recipients [20].

Extant studies in the OHP field have highlighted many factors in the nursing work environment that might increase the risk of developing burnout. It has been well-established that among human-service professionals, the most important burnout risk is represented by the emotionally demanding relationships between caregivers and recipients [26]. Additional work-related burnout-risk factors might encompass high job demands [27–29], experiencing role conflict [30], having to deal with a highly uncertain work environment [31], and perceiving poor social support or organizational justice [32].

However, existing studies also have highlighted extra-work events or conditions as burnout-risk factors. For instance, among health conditions responsible for influencing burnout, literature has provided evidence of musculoskeletal disorders [33] insomnia syndrome [34, 35], and depression [36]. On the other hand, no previous studies have focused on menopause, which is an important physiological transformation in female body balance. As recently pointed out by Hardy et al. [5], in the context of female employees at midlife, more research is needed to explore job stress in menopausal women, as well as the possible impact from menopausal symptoms on work outcomes. On the other hand, considering the high incidence of burnout among nurses in general and the high prevalence of women in this profession, it is essential to determine whether menopausal symptoms might contribute to the intensification of this risk in the context of nursing. Understanding the role of menopausal symptoms in contributing to burnout is crucial, especially given the well-known consequences of poor worker well-being on service quality and, thus, on service recipients’ well-being [37, 38].

Menopausal transition is a deep and pervasive process that entails changes in the physical, psychological, and cognitive spheres of women [3]; therefore, it is plausible that the associated symptomology might affect job burnout. For instance, several menopausal symptoms, such as a decrease in physical strength, difficulty sleeping, and fatigue might lead to workers having less psychophysical energy available for the job. In this view, a possible outcome might be an increase in emotional-exhaustion levels. Similarly, menopausal symptoms also might affect the quality of interpersonal relationships, including those with clients and patients. In a nursing context, psychosocial menopausal symptoms, including nervousness or irritability, might increase negative attitudes toward patients, fostering depersonalization. Moreover, according to preceding studies [39], women tend to report vasomotor symptoms as being the most difficult symptoms to manage in the workplace due to embarrassment and concern in relation to others in their midst.

Based on this, we propose the following hypotheses:

- H1: Menopausal symptoms are associated positively with emotional exhaustion.
- H2: Menopausal symptoms are associated positively with depersonalization.

### Moderating role of social and personal resources in the relationship between menopausal symptoms and burnout

The present study focuses on social and personal resources as possible buffers in the relationship between menopausal symptoms and burnout. Regarding social resources, based on work by Karasek and Theorell [40], we analyzed two principal aspects of social support in the workplace: support from colleagues and support from superiors concerning the levels of helpful social interaction available in the workplace from both.

As for personal resources, they refer to people’s sense of having control over their environments, including the ability to influence them [25, 41]. According to the psychological capital model perspective [42], it is possible to identify three principal personal resources relevant for work life: self-efficacy, resilience, and optimism. Self-efficacy is defined as an individual’s conviction (or confidence) about his or her abilities to mobilize the motivation, cognitive resources, and courses of action needed to execute a task successfully. Optimism refers to internal, relatively stable, and global attribution regarding positive events, such as goal achievement. Finally, resilience is characterized by positive coping and adaptation abilities to face significant adversity or risk, as well as recuperate after failure.

Both social and personal resources were found to be central in reducing stress in the workplace. For example, a large body of extant literature, mostly in the OHP field, has found that these resources play a buffering role against detrimental effects from various kinds of work-related stressors on job well-being outcomes [43, 44].

Ascertaining whether these resources work as moderators of the process that leads to burnout due to menopausal symptoms might help organizations identify proper actions at the individual and social levels that help women deal with their menopausal transitions at work.

No extant studies have tested the buffering effect of social and personal resources on the relationship between menopausal symptoms and burnout, but empirical evidence suggests plausibility in such a hypothesis. For instance, social support in one study was found to be a protective factor in the well-being of workers who returned to work after hospitalization [45]. On the other hand, other studies have demonstrated how both social and personal resources can buffer psychological well-being from adverse health-related events and their potential negative effects [46–49].

Based on this, we propose the following hypotheses:

- H3: Social and personal resources moderate the positive relationship between menopausal symptoms and emotional exhaustion, i.e., the relationship between menopausal symptoms and exhaustion is stronger in environments with few resources and weaker in environments with many resources.
- H4: Social and personal resources moderate the positive relationship between menopausal symptoms and depersonalization, i.e., the relationship between menopausal symptoms and depersonalization is stronger in environments with few resources and weaker in environments with many resources.

## Method

### Data collection and participants

The present study was developed within an agreement set out between the department of Psychology of the University of Turin and two Public Hospitals of the Piedmont region system (Italy), as a part of a broader research project aimed at assessing the quality of working life and work-related stress. The study design was cross-sectional and non-randomized. All the nurses employed in the two hospitals were asked to respond to a self-report questionnaire. The questionnaire included various scales directed at capturing the perceptions regarding the quality of working life and at assessing the nurse job-related well-being (e.g., burnout, social and personal resources). In the last pages, the questionnaire encompassed a section specifically dedicated to examine the link between well-being at work and menopause, to which only women in menopause were asked to respond (filter question: “are you in menopause”?). Data were collected in July–September 2016 and the self-report questionnaires were distributed during working hours. Participation in the survey was voluntary. To protect respondents’ confidentiality, workers were asked to enclose the completed questionnaire in an envelope and leave it in a case that the research team placed in each hospital.

The research conforms with 1964 Declaration of Helsinki provisions (and subsequent revisions), and all ethical guidelines were followed as required for conducting human research, including adherence to the legal requirements in the nation (Italy) where the study was conducted [50].

### Measures

The questionnaire included socio-demographic information (i.e., gender, age, job seniority) and sub-scales for measuring study variables (i.e., menopausal symptoms, social and personal resources, burnout, and work ability).

#### Menopausal symptoms

These were assessed using the Menopause-Specific Quality of Life (MENQOL [51]) questionnaire, which is self-administered and comprises 29 items (e.g., “experiencing hot flashes”). Items assess four main types of menopausal symptoms: vasomotor, psychosocial, physical, and sexual. Each item is rated as present or not present, and if present, how bothersome the items is on a scale of 0 (not bothersome) to 6 (extremely bothersome). Only women who responded positively to the question “Are you in menopause?” (response choices: “yes” or “no”) were asked to complete the MENQOL.

As no measure to assess menopausal symptoms was available in Italian, the original scale of MENQOL, one of the most used instrument in the literature, was adapted for an Italian context. In particular, following the International Guidelines on Test Adaptation [52], the original scale was translated into Italian by a member of the research group (Prof. Daniela Converso), reviewed and approved by all other research group members. The MENQOL was then back translated by an English native speakers. The two versions thus obtained were compared, discussed, and reviewed until a complete agreement was reached among the translator and the researchers. On the dataset obtained from the present survey, principal component analysis was used to explore factorial structure. The four-factor solution was not supported, though results supported a mono-dimensional solution in which all items significantly loaded (factor loading values fell between .41 and .84) on this one factor (13.88% of variance explained). In view of this finding, in the present study, MENQOL was treated as a single scale.

#### Outcome

Job burnout was measured through two sub-scales from the Maslach Burnout Inventory (original version: 26: Italian version [53]:): emotional exhaustion (nine items, e.g., “I feel emotionally drained from my work”) and depersonalization (five items, e.g., “I feel I treat some patients as if they were impersonal objects”). Responses on these scales were given on a four-point scale, ranging from 0 (never) to 6 (every day).

#### Social resources

Support from colleagues (five items, e.g., “People I work with are competent in doing their jobs”) and support from superiors (four items, e.g., “My supervisor is helpful in getting the job done”) were measured using two subscales from the Job Content Questionnaire (original version [54]:, Italian version [55]:). Responses on these scales were given on a four-point scale, ranging from 1 (not true) to 4 (completely true).

#### Personal resources

Self-efficacy (e.g., “At work, I’m able to manage any emergency and deal with unexpected tasks,” α =0.74) comprised five items and was measured using a scale developed by Caprara (scale originally developed in Italian [56]:). Optimism (e.g., “Even when facing work hardships, I expect things to turn out for the best”) comprised seven items and was developed by Carver et al. (original version [57]:, Italian version [58]:). Resilience (e.g., “At work, I am able to adapt to any change required by the situation”) comprised 10 items and was developed by Campbell-Sills et al. (original version [59]:, Italian adaptation [58]:). Responses on these scales were given on a four-point scale, ranging from 1 (not true) to 4 (completely true).

#### Control variables

Age and work ability [60] (measured with the Italian version of Work Ability Index [61, 62]: were included as control variables since it is recognized that they might work as potential confounders in studies that aim to identify burnout correlates [19, 62].

All sub-scales reported good internal consistency (see Table 1).

**Table 1:** Pearson’s correlations between study variables

	M (sd)	α	1	2	3	4	5	6	7	8	9	10
1. Exhaustion	12.78 (7.86)	.90	1									
2. Depersonalization	6.30 (6.90)	.76	.69^**^	1								
3. MENQOL	3.21 (1.36)	.93	.54^**^	.41^**^	1							
4. Support from superiors	2.46(.60)	.76	−.48^**^	−.25^*^	−.23^*^	1						
5. Support from colleagues	3.48(.89)	.85	−.25^*^	−.03	−.21	.48^**^	1					
6. Self-efficacy	2.96(.47)	.83	−.26^*^	−.18	−.15	.51^**^	.35^**^	1				
7. Optimism	2.62(.40)	.66	−.26^*^	−.26^*^	−.33^**^	.19	.10	.28^**^	1			
8. Resilience	2.81(.44)	.85	−.39^**^	−.35^**^	−.44^**^	.33^**^	.08	.30^**^	.42^**^	1		
9. Work ability	35.77 (5.87)	.78	−.47^**^	−.40^**^	−.46^**^	.34^**^	.20	.39^**^	.33^**^	.47^**^	1	
10. Age	53.56 (7.17)	–	−.02	−.05	−.06	−.02	−.09	.13	−.01	−.00	−.23^*^	1

Note: * *p* ≤ .05; **p ≤ .001

### Data analyses

Data analyses were performed using SPSS Statistics 25. Preliminary analyses included means, standard deviations, and Pearson correlations. To examine the moderating role of resources between menopausal symptoms and burnout (i.e., exhaustion and depersonalization), several moderated hierarchical regressions were performed.

For each moderated hierarchical regression, independent variables were entered in two successive steps. In the first step, the standardized indices of menopausal symptoms and a resource, as well as the interaction term (i.e., the product between menopausal symptoms and the resource considered), were entered. In the second step, control variables were entered (i.e., age and work ability). In cases in which the interaction term showed significant value, post-hoc analysis recommended by Aiken and West [63], consisting of a simple-slope test, was carried out to further probe that the association between the independent variable and the outcome is conditional on the value of the moderator.

To ensure that the dataset was sufficiently powered for a regression analysis, we calculated the a-priori sample-size using a software developed by Soper [64]. In the computation we assumed an anticipated effect size at .10, probability at .05, and power level of .80 [64]. At Step 1 of the regression analysis, where 3 independent variables were included, the minimum sample size was 76. At step 2, in which 5 independent variables were included, the minimum sample size was 91. Therefore, the sample of 94 nurses included in this study can be expected to have sufficient power to conduct the regression analyses above described.

## Results

### Descriptive analyses

In total, 524 nurses were contacted, and 333 questionnaires were returned to the research team (response rate: 63.54%). Of the 333 questionnaires returned to the research team, 276 were from women, and among those, 94 indicated menopause status.

In the present study, only the subsample of 94 women in menopause, which have completed the section in the questionnaire regarding work and menopause, was used.

The average age and job seniority (in years) in this subsample were 53.56 (sd = 7.17) and 27.16 (sd = 9.09), respectively. Among the major study variables, for work ability and burnout only were available in the literature well-established cut-off to categorize the score obtained. The average emotional exhaustion and depersonalization rates were 12.78 and 6.30, respectively. According to the manual of the Italian version [52], whereas exhaustion level score fell within the low category (≤3 = low; 4–8 = moderate; ≥9 = high), depersonalization score was within the moderate category (≤14 = low; 15–23 = moderate; ≥24 = high). The average work-ability rate was 35.77 (5.87), which fell within the “moderate” category of the Work Ability Index (7–27 = poor; 28–36 = moderate; 37–43 = good; 44–49 = excellent) [59].

### Preliminary analyses

Table 1 reports univariate relationships between variables under study. Menopausal symptoms were correlated positively with both emotional exhaustion and depersonalization. Exhaustion and depersonalization were found to be significantly and negatively associated with the resources considered, with two exceptions: Depersonalization did not correlate significantly with support from colleagues and self-efficacy.

Regarding control variables, both exhaustion and depersonalization were negatively associated with work ability, but not significantly associated with age.

### Moderated regression analyses

Table 2 reports the results of the moderated hierarchical regressions, in which emotional exhaustion was entered as the dependent variable. In the first step, all the models reported significant R^2^ and showed a variance explained that ranged from 30% (Model 1: support from superiors) to 36% (Model 3: self-efficacy). Regarding main effects, menopausal symptoms were found to be significant in all models (confirming H1). Among resources, only self-efficacy was negatively associated with exhaustion.

**Table 2:** Moderated regression analyses with exhaustion as the dependent variable

Step		1: superior support		2: colleague support		3: self-efficacy		4: optimism		5: resilience	
		β	p	β	p	β	p	β	p	β	p
1	MENQOL	.47	.000	.49	.000	.52	.000	.42	.001	.39	.001
	Resource	−.18	.079	−.18	.074	−.28	.009	−.13	.238	−.17	.129
	MENQOL x resource	.01	.913	−.17	.088	−.22	.036	−.17	.149	−.19	.069
2	MENQOL	.47	.003	.51	.000	.51	.000	.26	.054	.30	.010
	Resource	−.17	.229	−.14	.146	−.22	.027	−.10	.334	−.10	.383
	MENQOL x resource	−.05	.680	−.03	.723	−.09	.370	−.18	.104	−.17	.093
	Work ability	−.07	.012	.34	.001	.32	.001	−.32	.010	−.28	.019
	Age	−.00	.947	.02	.804	.04	.627	−.01	.879	.00	.996
1	R^2^	.30^**^		.36^**^		.36^**^		.34^**^		.35^**^	
2	R^2^	.37^**^		.45^**^		.45^**^		.41^**^		.41^**^	

Note: ***significant at .001

The interaction effect between menopausal symptoms and the resource was found to be significant in one model only: Self-efficacy buffered the effect of menopausal symptoms on emotional exhaustion. However, this finding was not confirmed in Step 2, when the model was adjusted for controlling variables (H3 was not confirmed).

Concerning control variables, work ability showed a significant and negative association with emotional exhaustion in all five models carried out. On the other hand, no significant value was found to be associated with age.

Table 3 shows the results for depersonalization. In the first step, all the models reported a significant R^2^. Menopausal symptoms were found to affect depersonalization significantly and positively in all models, excluding Model 4 and Model 5. Regarding the main effect of resources, support from superiors, support from colleagues, optimism, and resilience showed a significant association with depersonalization.

**Table 3:** Moderated regression analyses with depersonalization as the dependent variable

Step		1: superior support		2: colleague support		3: self-efficacy		4: optimism		5: resilience	
		β	p	β	p	β	p	β	p	β	p
1	MENQOL	.21	.048	.29	.009	.35	.003	.15	.259	.19	.103
	Resource	−.31	.003	−.24	.025	−.14	.210	−.27	.026	−.20	.094
	MENQOL x resource	−.32	.002	−.29	.008	−.15	.185	−.32	.011	−.36	.001
2	MENQOL	.14	.210	.14	.256	.20	.099	−.04	.765	.12	.331
	Resource	−.26	.014	−.19	.067	−.05	.687	−.24	.034	−.15	.201
	MENQOL x resource	−.32	.002	−.31	.003	−.20	.065	−.37	.002	−.35	.001
	Work ability	−.20	.086	−.31	.016	−.34	.015	−.34	.008	−.23	.060
	Age	.05	.595	.02	.837	.02	.886	.00	.984	.02	.804
1	R^2^	.35^**^		.28^**^		.19^**^		.27^**^		.32^**^	
2	R^2^	.39^**^		.36^**^		.28^**^		.36^**^		.37^**^	

Note: **significant at .001

**Fig. 1:**
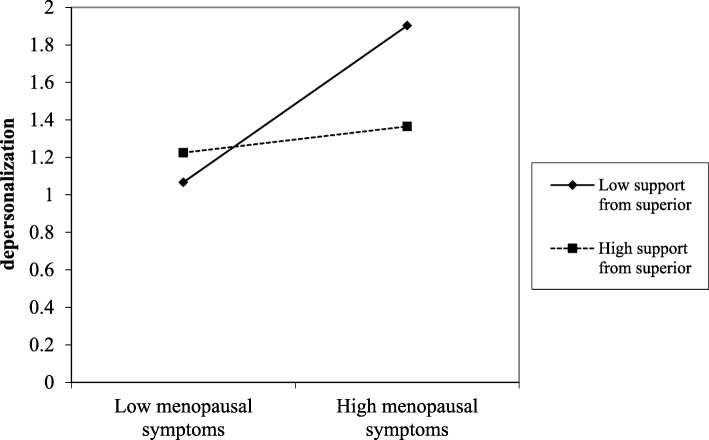
The effect of support from superior in the relationship between menopausal symptoms and depersonalization

**Fig. 2:**
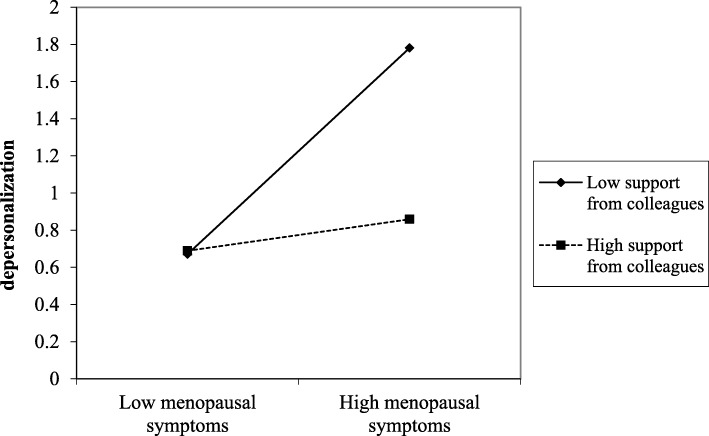
The effect of support from colleagues in the relationship between menopausal symptoms and depersonalization

**Fig. 3:**
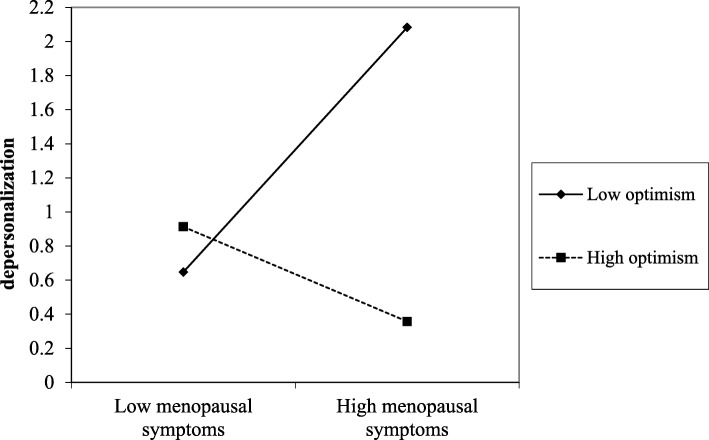
The effect of optimism in the relationship between menopausal symptoms and depersonalization

**Fig. 4:**
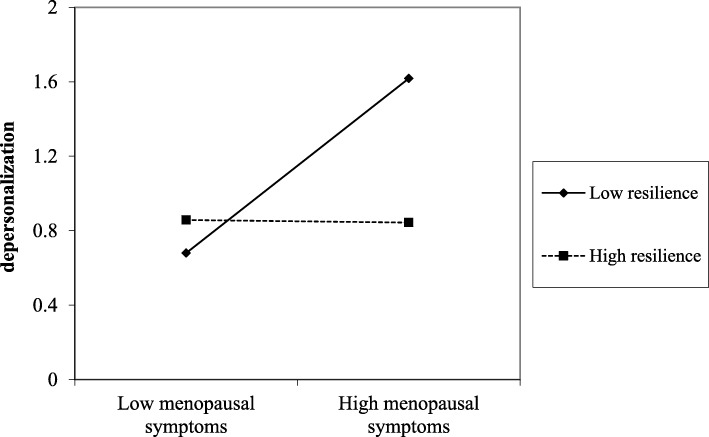
The effect of resilience in the relationship between menopausal symptoms and depersonalization

Regarding the interaction effect, it was found to be significant in four of the five models carried out, indicating that support from superiors, support from colleagues, optimism, and resilience moderated the detrimental effects from menopausal symptoms on depersonalization. The significance of these interaction effects were all observed also in Step 2, after adjusting the models for control variables. The plots of the significant interactions were reported in Figs. 1, 2, 3, and 4.

Results were supported by slope-test analysis. As reported in Table 4, in the case of high social (i.e., support from superior and colleagues) and personal (i.e., optimism and resilience) resources, the association between menopausal symptoms and depersonalization was not significant. On the other hand, in the case of low social (i.e., support from superior and colleagues) and personal (i.e., optimism and resilience) resources, the relationship between menopausal symptoms and depersonalization was positive and significant (H2 and H4 partially confirmed).

**Table 4:** Slope test on the significant interaction effects

	High resource	Low resource
MENQOL * Support from superior (Fig. 1)	β = .04; t = .21; *p* = .834	β = .49; t = 3.39; *p* = .001
MENQOL * Support from colleagues (Fig. 2)	β = .08; t = .46; *p* = .645	β = .55; t = 3.44; *p* = .001
MENQOL * Optimism (Fig. 3)	β = −.48; t = −1.69; *p* = .091	β = .52; t = 3.43; *p* = .001
MENQOL * Resilience (Fig. 4)	β = −.01; t = −.04; *p* = .969	β = .47; t = 3.03; *p* = .003

## Discussion

The aim of the present study was to examine the relationship between menopausal symptoms and job burnout in a sample of women during menopause. In particular, we assessed whether menopausal symptoms affected two burnout sub-dimensions, i.e., emotional exhaustion and depersonalization. In addition, we tested whether these relationships vary as a function (i.e., moderation) of any social (i.e., support from peers, support from superiors) or personal (i.e., efficacy, optimism, and resilience) resources.

Menopausal symptoms were found to be positively and strongly associated with emotional exhaustion. This relationship was significant, including after controlling for resources or confounding variables considered in our study (i.e., work ability and age). On the other hand, no personal or social resources moderated the detrimental effects of menopausal symptoms on nurse exhaustion.

According to Pearson’s correlations, depersonalization showed a significant, but weaker, if compared with emotional exhaustion, association with menopausal symptoms. In two multiple regressions, when a resource and the interaction term were included in the model, the relationship between menopausal symptoms and burnout stopped being significant (i.e., resilience and optimism). After including control variables, in all the models, this relationship stopped being significant. However, in four of the five models carried out, the interaction term indicated that the relationship between menopausal symptoms and depersonalization was significant – with the exception of self-efficacy – only when resources were low.

The present study highlighted that, despite being aspects of the same phenomenon (i.e., burnout), emotional exhaustion and depersonalization were affected differently by menopausal symptoms. Even though the relationship with menopausal symptoms and these two burnout dimensions was not previously tested, this finding is not completely unexpected. For instance, previous studies identified important differences between these two dimensions when examined in relation to the perception of the work environment [65]. Jourdain and Chenevert [66] found, in a sample of nurses, that job demands predicted emotional exhaustion, but not depersonalization. This finding can be explained through the differing natures of the two burnout dimensions. Emotional exhaustion is the energy component of burnout, referring to the end stage in a process of energy depletion. Depersonalization represents the motivational component of the syndrome, describing the relationship between the worker and the recipients of his or her job (i.e., patients, clients, or customers) in terms of involvement and (dis) engagement [25, 66].

The finding concerning the strong association between menopausal symptoms and exhaustion is in accordance with the large existing body literature, which has highlighted that lack of energy and fatigue feelings may represent important issues during menopause as these symptoms may seriously compromise women quality of life [67–69]. Moreover, this finding confirmed what the few pioneering studies, specifically carried out in the workplace, suggested, i.e., menopausal symptoms tend to activate a process of energy depletion, which might lead menopausal women to feeling exhausted at work [25]. In particular, those studies [13, 14, 16], have highlighted the urgency of considering the consequences of the psychophysical fatigue during menopause on the quality of the women work life, in particular concerning the question of work-related stress. A previous study [70], examining the relationship between stress and fatigue, has highlighted that the dynamic relationship between these two variables change dramatically during menopausal transition. According to the authors [70], this change can be attributable to a dysregulation in the general homeostatic equilibrium of the body, which may lead, in turn, to a decrease of the ability to bounce back from either stress or fatigue. In the same direction, our findings shed light on the very deep and pervasive nature of this process of depletion of psychophysiological energy at work during menopause. First, after including control variables, the effect of menopausal symptoms kept being significant. Secondly, none of the resources considered was found to moderate this relationship. The findings that both personal and social resources did not moderate the relationship between menopausal symptoms and exhaustion can be explained considering that those resources might not represent effective “tools” to help manage fatigue, because they do not offer specific opportunities to prevent exhaustion, for example by facilitating the energy recovery process [71]. This interpretation is in particular suggested by the matching principle of the Demand-Induced Strain Compensation (DISC) model [72], which proposes that the stress-buffering effect of resources occurs more often when stressors, resources, and outcomes belong to the same domain. Accordingly, rather than social or personal resources, job resources which give the opportunity to recover the energy depleted, such as such as work flexibility or autonomy on the job, might moderate the relationship between menopausal symptoms and exhaustion [73]. However, even if fascinating, this interpretation, at the current state-of-art of the knowledge, remains a speculation. In fact, no previous empirical evidence is available regarding possible moderators of the relationship between menopausal symptoms and any work-related well-being outcomes. Therefore, it is vital that this line of research will be further expanded. In particular, future studies could test whether other aspects of the work environment, such as work flexibility or autonomy on the job, might buffer these relationships by giving women instruments to recover energy, when needed, to maintain the balance between work and health [4, 74]. Generally speaking, given the strong relationship between menopausal symptoms and exhaustion, it is important for future research to focus on the moderators of this relationship, as the potential knowledge gained might support the development of proper actions and interventions directed at helping women deal with their menopausal transition.

On the other hand, the effect of menopausal symptoms on depersonalization was found to be weaker. In particular, the detrimental effect of menopausal symptoms on depersonalization seems to be activated by poor personal and social resources. On the contrary, having high personal and social resources was found to help women better manage menopausal transition, avoiding negative implications for recipients of their services. As suggested by Bariola and colleagues [15], it is possible that receiving support from colleagues and superior in managing patients, especially in condition of criticality (e.g., aggressive patients), may represent a resource that allow women, despite the presence of menopausal symptoms, to preserve motivation to manage effectively and with empathy the relationship with their patients. These results are also in accordance with the matching principle of the DISC Model [72, 73], indicating that social resources (i.e., co-worker support) are effective in buffering the effect of menopausal symptoms on a similar outcome, i.e., depersonalization describes negative attitudes toward patients.

Moreover, it is plausible that high levels of resilience and optimism may help contrast certain menopausal symptoms in particular, mood irritability or anxiety [75]. This may help, in turn, to minimize the development of negative attitudes that negatively impact on the relationship with recipients. The only resource which was found not to moderate the association between menopausal symptoms and depersonalization is self-efficacy. This finding suggest that each resource works differently in moderating this relationship. Therefore, future studies should be aimed at examining the role of other job resources in buffering the effect of menopausal symptoms on burnout.

In general, the role of personal and social resources in contrasting menopausal symptoms has been previously recognized [17, 76]. However, the present study has advanced the literature by contributing to shed light on the specific mechanisms by which this resource may interrupt link between menopausal symptoms and depersonalization. This result is particularly important considering the negative consequences of depersonalization, highlighted by previous studies, in terms of diminished quality of service [38], increased turnover [77], and absenteeism [78].

However, the present study is not without limitations. First, the small sample size might have reduced the study’s power by increasing the margin of error. Future studies should examine larger numbers of menopausal women.

Another limitation is the cross-sectional design. Future research should employ longitudinal studies to validate cross-sectional findings obtained in the present study, in order to ascertain that it is menopausal symptoms that lead to increase burnout, not vice versa.

A further limitation is that no instrument adapted for an Italian context to measure menopausal symptoms was available. Considering this gap, the research group proceed to translate the MENQOL, one of the most used instrument in the literature, from English to Italian. Preliminary exploratory analyses regarding psychometric proprieties were carried out. However, given the small size of the sample in the current study, further studies focused on the examination of the psychometric proprieties are needed, in order to validate the Italian version of MENQOL here used.

Moreover, all the measures employed were self-reported. Data coming from a single source might introduce the issue of common method variance [79]. Future studies might benefit from employing research designs that include a combination of objective and subjective measures or using data from multiple sources (e.g., the inclusion of a medical assessment for menopausal symptoms).

Finally, the use of a non-randomized sample represents a limiting factor for this study and some biases might have affected our study findings. For example, nurses who perceived lower level of quality of working life, might have been more motivated to participate to the survey. Therefore, caution should be exercised when generalizing the results to other nursing populations.

## Conclusions

These results hold important practical implications. For menopausal women, relying on superiors and colleagues who consider their needs as workers and as people represents an important resource to help maintain a positive relationship, including with their service recipients. In this view, the present study suggests that interventions aimed at improving social climate are crucial. These types of interventions might support not only all workers in dealing with job demands as previously demonstrated [80], but also menopausal women who are dealing with a transition that might be stressful and disabling. Examples of interventions in this direction include training directed at developing managerial skills among nurse coordinators. In addition, team-building interventions that aim to encourage teamwork also might be beneficial. On the other hand, having many personal resources might help contain depersonalization among menopausal women. In this view, actions specifically directed at increasing personal resources among menopausal women, such as offering mindfulness classes [81] or psychological support services, might benefit not only menopausal women’s psychological health, but also the quality of service.

## Data Availability

Dataset supporting the conclusions of this article are available and can be requested from the corresponding author.

## Abbreviations

MENQOL: Menopause-Specific Quality of Life
OHP: Occupational Health Psychology
